# Clinical implications of the BRAF mutation in papillary thyroid carcinoma and chronic lymphocytic thyroiditis

**DOI:** 10.1186/s40463-017-0247-6

**Published:** 2018-01-09

**Authors:** Woon Won Kim, Tae Kwun Ha, Sung Kwon Bae

**Affiliations:** 1 Department of General Surgery, Haeundae Paik Hospital, Inje University College of Medicine, Busan, Republic of Korea; 2 Department of General Surgery, Busan Paik Hospital, Inje University College of Medicine, 75, Bokji-ro, Busanjin-gu, 614-735 Busan, Republic of Korea; 3 Department of Medical Management, Kosin University, Busan, Republic of Korea

**Keywords:** BRAF mutation, Chronic lymphocytic thyroiditis, Papillary thyroid carcinoma

## Abstract

**Background:**

The purpose of this study was to examine the possible prognostics and clinicopathologic characteristics underlying the BRAFV600E mutation and papillary thyroid carcinoma (PTC) coexisting or in absence of chronic lymphocytic thyroiditis (CLT).

**Methods:**

This study was conducted on 172 patients who had undergone total thyroidectomy or unilateral total thyroidectomy for PTC; the patients were then examined for the BRAFV600E mutation using specimens obtained after their surgery from January 2013 to August 2015.

**Results:**

BRAF mutations were found in 130 of 172 patients (75.6%). CLT was present in 27.9% of patients (48/172). The incidence of the BRAFV600E mutation was significantly increased in the group with no CLT (*P* = 0.001). The findings of the multivariate analysis pertaining to the coexistence of CLT and PTC showed no significant correlation other than the BRAFV600E mutation. No significant difference was noted in the clinicopathologic factors between the two groups based on the coexistence of CLT in univariate and multivariate analyses.

**Conclusions:**

The BRAFV600E mutation is less frequent in PTC coexisting with CLT presumably because CLT and the BRAFV600E mutation operate independently in the formation and progression of thyroid cancer.

## Background

Papillary thyroid carcinoma (PTC) is the most common type of thyroid cancer and accounts for approximately 80- 85% of malignant neoplasms of the thyroid gland. It has a 10-year survival rate 93% in the United States, which is relatively favorable compared to that of malignant tumors [1]. The major factors underlying the prognosis of PTC include patient age, gender, tumor size, histological findings, extrathyroidal extension, clinical lymph node metastasis and remote metastasis [2]. BRAF gene mutations have also been reported to be factors that can most accurately predict lymph node metastasis, extrathyroidal extension, advanced disease stages III and IV, and disease recurrence [3]. Among the various isoforms of Raf kinase, the B-type RAF V600E (BRAFV600E) mutation is the most commonly observed genetic abnormality in PTC that induces excessive proliferation and differentiation of tumor cells at the initial tumor stage. It is involved in, not only tumorigenesis but also in the conversion to aggressive, non-differentiated cancer [4].

Chronic lymphocytic thyroiditis (CLT) is a common autoimmune thyroid disorder in which the thyroid gland is attacked by various antibodies and cell-mediated immune processes [5]. Dailey et al. reported the coexistence of CLT in thyroid cancer for the first time in 1955 [6]. The ratio of coexistence has varied between 10.7% and 27.6% in 2008, respectively [7]. The role that CLT plays as a prognostic factor in thyroid cancer is controversial, but it is known that PTC is approximately three times in the presence of CLT [8]. Recent studies have reported an association between Hashimoto's thyroiditis, the BRAFV600E mutation, and clinicopathologic features in patients with PTC [9]. PTC with coexisting CLT was less associated with extrathyroidal extension and lymph node metastasis; CLT antagonizes PTC progression when accompanying a positive BRAFV600E mutation [10]. However, this issue is still controversial as more studies are needed on the correlation between CLT and the BRAFV600E mutation as a prognostic factor for PTC. This study reviews the frequency of the BRAFV600E mutation in thyroid tissues extracted after surgery on the thyroid gland and examines the relationship between the BRAFV600E mutation and CLT as well as the correlation with prognostic factors to review clinicopathologic characteristics.

## Methods

This study was conducted on 172 patients who had undergone total thyroidectomy or unilateral total thyroidectomy for PTC; the patients were then examined for the BRAFV600E mutation using specimens obtained after surgery in the Busan Paik Hospital from January 2013 to August 2015. Our institutional review board approved this retrospective study and waived the need for informed consent based on the retrospective design. CLT is an autoimmune disease characterized by widespread lymphocyte infiltration, fibrosis and parenchyma atrophy of thyroid tissue. Pathologically proven CLT was defined as the presence of diffuse lymphocytic and plasma cell infiltrate, oxyphilic cells and the formation of lymphoid follicles or reactive germinal centers in the area of normal thyroid tissue [11]. To confirm the clinicopathologic differences in PTC in the presence or absence of CLT and the BRAFV600E mutation, the patient’s age and sex, size of tumor, multiplicity or bilaterality of the tumor, presence of extrathyroidal extension, cervical or lateral cervical lymph node metastasis, and TNM staging (AJCC 7^th^) were analyzed. If there were multiple tumors, the size of the largest tumor was measured. Regardless of the number of tumors, cases in which there was more than one malignant tumor present in both lobes were defined as bilateral.

### DNA extraction & detection of the BRAFV600E mutation

Genomic DNA was extracted from the tumor using the QIAmap DNA formalin fixed paraffin-embedded extraction kit (Qiagen, Hilden, Germany) according to the manufacturer’s instructions. The extracted DNA was measured by UV absorption (Nanodrop; Thermo Scientific, Wilmington, DE). For every specimen, a total of 41mg of genomic DNA was typically extracted. The T1799A mutation in BRAF exon 15 leads to a substitution of V for E at residue 600 in PTCs. The T1799A transversion was detected using the Anyplex BRAF V600E real-time detection system (Seegen Inc., Seoul, Korea). Real-time PCR was performed using a CFX96 real-time PCR system (Bio-Rad, Hercules, CA). The cycle threshold (Ct) of real-time PCR was defined as the number of amplification cycles at which the level of fluorescent signal exceeded the threshold for the presence of the BRAF mutation. This cut-off value was determined based on the average Ct value found in 100 repeats of low-positive concentrations of the BRAF^V600E^ plasmid DNA for which a positive rate of 100% was achieved. The Ct value of the target and the internal control was 533 and 30, respectively, and each run consisted of both positive and negative controls.

### Statistical analysis

SPSS Version 21(SPSS Inc., Chicago, IL, USA) was used for statistical analyses of the data. The univariate variable between each group was analyzed using the chi-square test or T-test, and the corrected univariate variables were analyzed using logistic regression analysis. The statistical significance level was set at *P*<0.05.

## Results

The mean age of the 172 PTC patients (20 males and 152 females) was 49.1±11.35 years (range 18–79), and the mean tumor size was 0.91 ± 0.72 cm (range 0.2–4.5 cm). Papillary thyroid microcarcinomas measuring <1 cm in size were observed in 124 cases (72.1%). The BRAFV600E mutation was confirmed in 130 PTC patients (75.6%). Metastasis in the central lymph node (N1a) was observed in 74 patients (43.0%), whereas metastasis in the lateral cervical lymph node (N1b) was confirmed in 18 patients (10.5%). Forty-eight of the 172 PTC patients (27.9%) had CLT, and the remaining 124 patients (72.1%) did not have CLT. Forty-three patients (25.0%) had multiple tumors on one lobe, whereas 33 patients (19.2%) had bilateral PTC. Of the 62 patients (36.0%) with extrathyroidal extension. Two patients showed airway, esophageal, or recurrent laryngeal nerve invasion. Overall, 106 patients (60 aged <45 years and 46 cases aged>45 years) had TNM stage 1, whereas 51 cases (29.7%) had TNM stage 3, and 14 (8.1%) had TNM stage 4a (Table 1).

**Table 1:** Demographic characteristics of 172 patients with papillary thyroid carcinoma

Characteristics	Value (%)
Total number	172(100.0)
Gender	
Female	152 (88.4)
Male	20 (11.6)
Age (year) at diagnosis (Mean ±S.D)	49.1±11.35
< 45	60 (34.9)
≥ 45	112(65.1)
Tumor size (mm)(Mean±S.D)	0.91 ± 0.47
1 cm	124(72.1)
≥ 1 cm	48(27.9)
Multifocality	
Unifocal	129 (75.0)
Multifocal	43 (25.0)
Bilaterality	
Unilateral	139 (80.8)
Bilateral	33 (19.2)
Extra thyroidal extension	
Absent	110 (64.0)
Present	62 (36.0)
Central lymph node metastasis	
Absent	98 (57.0)
Present	74 (43.0)
Lateral lymph node metastasis	
Absent	154 (89.5)
Present	18 (10.5)
Stage	
I	106(61.6%)
II	1 (0.6%)
III	51 (29.7)
IVa	14 (8.1)
Chronic lymphocytic thyroiditis	
Absent	124 (72.1)
Present	48 (27.9)
BRAF mutation	
Absent	42 (24.4)
Present	130 (75.6)

*S.D* Standard deviation

### Clinicopathologic factors depending on the presence of CLT in PTC

The patients with CLT were younger than those without CLT (*P* = 0.06), and the number of women was high but did not show a statistically significant difference (*P* = 0.171). No statistically significant difference was noted in the size of PTC, its multiplicity or bilaterality, extrathyroidal extension, or the frequencies of cervical and lateral cervical lymph node metastasis.

The incidence of the BRAFV600E mutation was observed significantly more in the group with no CLT (82.3%) (*P* = 0.001, Table 2).

**Table 2:** Clinicopathologic factors related to chronic lymphocytic thyroiditis in 172 patients with PTC

	CLT(-)		CLT(+)		Χ^2^/t-test	*P*-value
	Count	%	Count	%		
Gender						
Female	107	(13.7)	45	(6.3)	1.874	0.171
Male	17	(86.3)	3	(93.8)		
Age (Mean ±S.D)	50.06±11.51		46.44±10.62		1.893	0.060
< 45	39	(31.5)	21	(43.8)	2.304	0.129
≥ 45	85	(68.5)	27	(56.3)		
Tumor Size(mm)(Mean ±S.D)	0.92 ±0.73		0.88±0.69		0.383	0.702
< 1.0 cm	90	(72.6)	34	(70.8)	0.908	0.635
≥ 1.0 cm	34	(27.4)	14	(29.2)		
Multifocality						
Absent	94	(75.8)	35	(72.9)	0.154	0.695
Present	30	(24.2)	13	(27.1)		
Bilaterality						
Absent	101	(81.5)	38	(79.2)	0.117	0.733
Present	23	(18.5)	10	(20.8)		
Extrathyroidal extension						
Absent	76	(61.3)	34	(70.8)	1.367	0.242
Present	48	(38.7)	14	(29.2)		
Central LM metastasis						
Absent	66	(53.2)	32	(66.7)	2.550	0.110
Present	58	(46.8)	16	(33.3)		
Lateral LM metastasis						
Absent	110	(88.7)	44	(91.7)	0.323	0.570
Present	14	(11.3)	4	(8.3)		
TNM stage						
I & II	71	(57.3)	36	(75.0)	5.770	0.056
III	40	(32.3)	11	(22.9)		
IVa	13	(10.5)	1	(2.1)		
BRAF mutation						
Negative	22	(17.7)	20	(41.7)	10.732	0.001
Positive	102	(82.3)	28	(58.3)		

*LN* Lymph node, *TNM* Tumor/node/metastasis

### Multivariate analysis pertaining to the correlation between CLT and BRAFV600E mutation

Univariate analysis of the relationship between PTC and clinicopathologic factors in the presence and absence of CLT was conducted. According to the analysis, the presence of the BRAF mutation showed a noticeable interdependence. In the presence of the BRAF mutation, CLT was found at a lower frequency. This finding suggests that the presence of CLT has no association with BRAF- mutated PTC (*P*=0.001) (Table 2).

The gender and age variants, which appeared to potently influence the relationship between CLT and PTC, and the BRAF mutation were included in the logistic regression analysis. Only the BRAFV600E mutation had a statistically significant correlation with the coexistence of CLT and PTC in the multivariate analysis (*P*=0.002) (Table 3).

**Table 3:** Multivariate analysis of clinicopathologicfactors associated with the BRAF mutation in papillary thyroid carcinoma with or without CLT

	B	S.E	Sig.	Exp (B)	95% CI	
					Lower	Upper
Gender	-0.819	0.667	0.220	0.441	0.119	1.630
Age	0.501	0.363	0.167	1.651	0.810	3.363
BRAF	1.184	0.380	0.002	3.267	1.550	6.887
Constant	-1.398	0.266	0.000	0.247		

*B* Significance probability, *S.E* Standard error, *Sig* Significance probability

*Exp(B)* Odds ratio, *CI* Confidence interval

### Comparison of PTC cases with the BRAFV600E mutation based on the presence of CLT

Of 130 PTC patients with the BRAFV600E mutation, 28 (21.5%) had CLT. No significant difference was noted in the clinicopathologic factors between the two groups categorized based on the coexistence of CLT in univariate and multivariate analyses.

## Discussion

PTC is the most common thyroid cancer and gradually leads to remote metastasis, accounting for 88% of thyroid cancer cases. PTC has an overall favorable prognosis with an average 10-year survival rate of 93%, although up to 10% of patients eventually die as a result of the disease [12]. However, the frequency of coexisting CLT in patients with PTC has been reported to range from 0.5%-38% [13]. CLT is one of the autoimmune diseases that shrinks the thyroid parenchyma through infiltration or fibrosis and is the most common inflammatory thyroid disease with a frequency of 22 in 100,000 patients. CLT is 15–20 times more likely to occur in women and is more frequently seen in patients between the age of 30 and 50 years; however, it is observed in all age groups. CLT may exhibit no symptoms or a slight deterioration in thyroid function as a major cause of goiter but is not accompanied by pain. Our study shows a 27.9% coexistence between PTC and CLT, which is similar to previous studies. The correlation between the two diseases is still controversial. Some suggest that there is a positive correlation with CLT, as the activated inflammatory response present in CLT creates a favorable setting for malignant transformation. The inflammatory response may cause DNA damage through the formation of reactive oxygen species, resulting in mutations that eventually lead to the development of PTC. However, it is unclear whether CLT may lead to PTC in patients; or that CLT may be a secondary finding with PTC; or CLT and PTC may be part of a host tumor response system [14].

This study examined whether BRAFV600E is associated with CLT and PTC. BRAFV600E is only observed in anaplastic carcinoma originated from PTC or papillary carcinoma and not in other thyroid cancers, including follicular carcinoma. BRAF is a B-type Raf kinase located on chromosome 7 and is the most potent activator of the mitogen-activated protein kinase/extracellular-signal-regulated kinase (MEK-ERK) pathway. The most common hotspot mutation in the BRAF gene is a thiamine transversion to adenine at nucleotide position 1799 (T1799A) in exon 15. This transversion causes a conversion of valine to glutamate of amino acid 600 in the BRAF protein, creating a constitutively active BRAF kinase, which has been proven to be an oncogene in human cancer [12] and is found in 40%–80% of all cases of papillary thyroid cancer [15].

Many studies have reported correlations between the aggressive clinical factors underlying the BRAFV600E mutation and papillary carcinoma. According to Liu X et al., a multi-institutional research study suggested that extra-thyroidal extension, multifocality, lymph node metastasis, and advanced TNM were associated with the BRAFV600E mutation in PTC [16]. The BRAFV600E mutation has been reported to increase not only the aggressive nature of the tumor but also the recurrence and mortality rates of the disease [17]. These findings suggest the need for more proactive surgery or radioactive iodine therapies and more careful follow-up observations. However, its value as a prognostic precursor is still debated because some studies have shown contradictory findings on the correlation between the BRAFV600E mutation and the aggressiveness and poor prognosis of PTC [18].

This study showed a significantly low frequency of the BRAFV600E mutation in PTC coexisting with CLT. The frequency of the BRAFV600E mutation in PTC with and without CLT was 58.3% and 82.3%, respectively (*P* = 0.001). This correlation was independently confirmed through a multivariate analysis that was adjusted for sex and age(OR: 0.353, 95%CI: 0.148–0.842). This finding is consistent with previous research findings on the significantly low frequency of the BRAFV600E mutation in PTC coexisting with CLT [19]. This result implies that PTC with CLT has a developmental mechanism that is different from the molecular genetic background of the BRAFV600E mutation. However, some PTC groups with CLT showed higher frequencies of the BRAFV600E mutation, while other studies reported that a group with a positive BRAFV600E mutation was an independently good prognostic factor [9].

PTC coexisting with CLT is generally known to be common among women; it has a good prognosis at early stages and a low recurrence rate. Existing literature does not show whether the presence of thyroiditis alters the biologic effect of PTC. In this study, the presence of thyroiditis seemed to lessen the invasive potential of PTC. There was less extrathyroidal extension and less multifocality and bilaterality in PTC with CLT. The presence of thyroiditis with PTC was associated with less lymph node metastasis on presentation. The characteristics of the clinicopathologic factors in the presence or absence of CLT did not exhibit statistically significant differences. Therefore, we can infer that CLT and the BRAFV600E mutation do not mutually affect PTC generation and progress.

This study is limited in the following ways: 1) positively prognostic and non-aggressive papillary microcarcinomas <1 cm in size are very frequent; 2) the short timeline of research and data collection in this study prevents possible long-term relevant findings for prognoses. Long-term follow-up studies are also required, with a larger sample of patients and from multiple institutions.

## Conclusion

Given that the BRAFV600E mutation is less frequent in PTC with CLT, CLT and the BRAFV600E mutation presumably have independent mechanisms on how they affect the formation and progression of thyroid cancer.

## Abbreviations

CLT: Chronic lymphocytic thyroiditis,
PTC: Papillary thyroid carcinomas
BRAF: b-type raf kinase
TNM: Tumor/node/metastasis
DNA: Deoxyribonucleic acid
UV: Ultraviolet
PCR: Polymerase chain reaction
Ct: Cycle treshold
S.D: Standard deviation
LN: Lymph node
TNM: Tumor/node/metastasis
B: Coefficient of regression,
S.E: Standard error
Sig: Significance probability
Exp(B): Odds ratio
CI: Confidence interval
